# Crystal structure of AFV3-109, a highly conserved protein from crenarchaeal viruses

**DOI:** 10.1186/1743-422X-4-12

**Published:** 2007-01-22

**Authors:** Jenny Keller, Nicolas Leulliot, Christian Cambillau, Valérie Campanacci, Stéphanie Porciero, David Prangishvili, Patrick Forterre, Diego Cortez, Sophie Quevillon-Cheruel, Herman van Tilbeurgh

**Affiliations:** 1 Institut de Biochimie et de Biophysique Moléculaire et Cellulaire, CNRS-UMR 8619, Université Paris 11, IFR115, Bâtiment 430, 91405 Orsay, France; 2 Architecture et Fonction des Macromolécules Biologiques, CNRS and Universités d'Aix-Marseille I & II, UMR 6098, Case 932, 163 avenue de Luminy, 13288 Marseille cedex 9, France; 3 Institut Pasteur, Unité de Biologie Moléculaire du Gène chez les Extrêmophiles, 25, rue du Dr. Roux, 75015 Paris, France

## Abstract

The extraordinary morphologies of viruses infecting hyperthermophilic archaea clearly distinguish them from bacterial and eukaryotic viruses. Moreover, their genomes code for proteins that to a large extend have no related sequences in the extent databases. However, a small pool of genes is shared by overlapping subsets of these viruses, and the most conserved gene, exemplified by the ORF109 of the *Acidianus *Filamentous Virus 3, AFV3, is present on genomes of members of three viral familes, the *Lipothrixviridae, Rudiviridae*, and *"Bicaudaviridae"*, as well as of the unclassified *Sulfolobus *Turreted Icosahedral Virus, STIV. We present here the crystal structure of the protein (Mr = 13.1 kD, 109 residues) encoded by the AFV3 ORF 109 in two different crystal forms at 1.5 and 1.3 Å resolution. The structure of AFV3-109 is a five stranded β-sheet with loops on one side and three helices on the other. It forms a dimer adopting the shape of a cradle that encompasses the best conserved regions of the sequence. No protein with a related fold could be identified except for the ortholog from STIV1, whose structure was deposited at the Protein Data Bank. We could clearly identify a well bound glycerol inside the cradle, contacting exclusively totally conserved residues. This interaction was confirmed in solution by fluorescence titration. Although the function of AFV3-109 cannot be deduced directly from its structure, structural homology with the STIV1 protein, and the size and charge distribution of the cavity suggested it could interact with nucleic acids. Fluorescence quenching titrations also showed that AFV3-109 interacts with dsDNA. Genomic sequence analysis revealed bacterial homologs of AFV3-109 as a part of a putative previously unidentified prophage sequences in some Firmicutes.

## Background

Studies on viral diversity in geothermally heated aquatic environments, at temperatures above 80°C, resulted in isolation of about two dozens of double-stranded DNA viruses infecting members of the third domain of life, the Archaea [1]. The viruses have diverse unusual morphotypes, not encountered among dsDNA viruses of the Bacteria or Eukarya. Moreover, the detailed analysis of their genomes led to the conclusion that hyperthermophilic archaeal viruses are evolutionarily unrelated to other known viruses, and form a singular group in the viral world with a unique origin, or more likely, multiple origins [2]. Based on morphological and genomic characteristics, these viruses have been assigned to seven novel viral families: stiff, rod-shaped Rudiviridae [3], filamentous Lipothrixviridae [4-7], spindle-shaped Fuselloviridae [8-10], droplet-shaped SNDV [11], spherical "Globuloviridae" [12,13], bottle-shaped "Ampullaviridae" [14], and two-tailed "Bicaudaviridae" [15]. Three more hyperthermophilic archaeal viruses, the icosahedral STIV [16], spindle-shaped STSV1 [17] and PSV [18] have still not been classified.

A most prominent feature of the genomes of hyperthermophilic archaeal viruses is an extremely low number of genes coding for proteins homologous to any sequences in the existing sequence databases, be it proteins of other viruses or those of cellular life forms [2]. A few encoded proteins, functions of which have been recognised and confirmed biochemically, include the dUTPase [3] and the Holliday junction resolvase [19] of the rudiviruses SIRV1 and SIRV2 and the integrase/recombinase of the fusellovirus SSV1 [20]. The viruses from different families share a very small pool of genes with putative functions, including predicted transcription regulators, glycosylases, ATPase, as well as small proteins of unknown function from an uncharacterized YddF family [2]. The later protein family has three bacterial representatives in *Bacillus subtilis*, *Clostridium beijerincki*, and *Alkaliphilus metalliredigenes*, with all other members found specifically in archaeal viruses: members of the families *Rudiviridae, Lipothrixviridae*, *Bicaudaviridae *and the unclassified STIV [2]. A general presence of this protein in the members of the family Lipothrixviridae has been recently confirmed by its identification in the novel member of this family, the virus AFV3 [7]. The strong conservation of this protein in otherwise very different virus families suggests it may play an important function.

The power of structural analysis in establishing evolutionary relation ships among viruses was recently demonstrated by the structure determination of the major capsid protein of the Sulfulobus turreted icosahedral virus [21] and by the identification of a potential glysolyl transferase in the same virus [22]. This structure identified a common fold with capsid proteins from eukaryotic, bacterial and mammalian viruses. In absence of sequence similarity with proteins of known function, three dimensional structure is often an efficient approach to bring up strong hypothesis about protein function. With these observations in mind we decided to embark upon a systematic structure determination of crenarchaeal viral proteins [23]. In light of the small sequence resemblance with other proteins, we considered that these organisms might be enriched in novel folds. Secondly, in order to better understand the mechanisms of infection and the very peculiar morphologies, the biochemical function of these proteins must be investigated. We present here the 3D structure of an ORF [gid:3174] product from AFV3, named AFV3-109 as a first result of our archaeal virus structural proteomics project. AFV3-109 possesses a unique fold, close to that of the B116 protein, a STIV ortholog of unknown function whose structure was recently deposited at the PDB (code 2BLK). A glycerol molecule bound to a totally conserved surface patch may be a useful observation for further experimentation. We also found experimental evidence that AFV3-109 binds DNA.

## Results and discussion

### Overall structure

**Figure 1:**
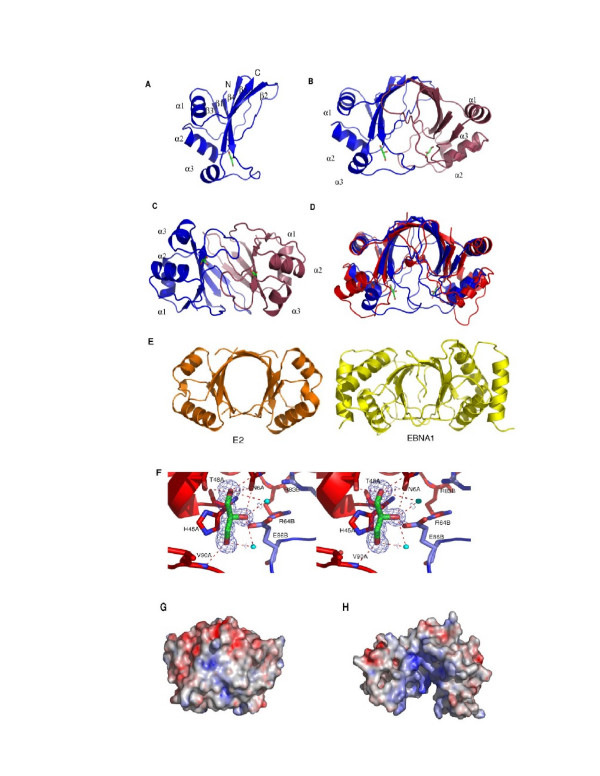
Structure of AFV3-109 monomer and dimer. A) Monomer presentation of AFV3-109. Secondary structure elements are labelled according to Figure 2. B,C) Two perpendicular representations of the AFV3-109 dimer (monomers are coloured bleu and deep red). Bound glycerol is shown as in green and red sticks. D) Superposition of AFV3-109 (blue) and B116 (red) dimers highlighting the conformational difference of the β4β5 connexion in both proteins. E) Architectural similarities between AFV3-109 and eukaryotic viral transcriptional regulators: ribbon representation of papilloma virus E2 (PDB code: 1JJH) and EBNA1 Epstein Barr Virus (PDB code: 1VHI) dimers. F) Stereo view of the 2fo-fc residual density in front of the conserved His45, modeled by a glycerol moiety (in sticks). Hydrogen bonds are represented by dashed lines, bound waters as spheres. Residues from both monomers are labeled A and B. G) Electrostatic potential surface of the AFV3-109 dimer (same orientation as in A). H) Electrostatic potential surface of an 'open' configuration of the AFV3-109 dimer, modeled on the B116 dimer structure, revealing a strongly positively charged surface inside the cavity.

We have overexpressed and purified AFV3-109 as a his-tagged fusion protein. The protein was crystallized in two different space groups, C222_1 _and P3_1_21, depending on the pH of the mother liquor (pH 8.8 and 4.0, respectively). The structures were solved at 1.5 (C222_1_) and 1.3 Å (P3_1_21) resolution. The statistics on data collection and refinement are provided in Table 1. Crystals of both space groups contain a single copy of the protein in the asymmetric unit. Both structures are almost identical with a r.m.s. deviation between both crystal forms of 0.5Å for all Cα positions. The AFV3-109 core is formed by a mixed five stranded curved β-sheet with the first strand perpendicular to the last (Figure 1a). The strand order is β3β1β4β5β2 with β5 anti parallel to the others. Helices and loops are providing cross-over connections between the strands. These connections are segregated on either side of the central β-sheet: all loops are situated on one face, while the helices cover a large part of the other. The three helices (α1 connecting β2β3, α2 connecting β3β4 and α3 connecting β4β5) are perpendicular to each other and establish only a few hydrophobic contacts between them.

**Table 1:** Data collection and refinement statistics

	(SeMet peak)	native	native
Space group	P3_1_21	C222_1_	P3_1_21
Unit-cell parameters	77.31 77.31	82.77 83.53	77.56 77.56
***a, b, c ***(Å)	37.07	33.99	37.31
Resolution (Å)	2.0 (2.11–2.00)	1.5 (1.58–1.50)	1.3 (1.33–1.30)
Total number of refl.	113230 (1638)	101423 (3139)	315160 (12016)
Total of unique refl.	8855 (1260)	16423 (490)	31813 (1183)
Multiplicity	12.8 (9.1)	5.9 (5.5)	9.9 (8.6)
R_merge_^1^	0.061 (0.032)	0.052 (.024)	0.145 (0.107)
I/σ(I)	31.4 (11.5)	19.2 (3.9)	13.1 (2.8)
Overall completeness (%)	99.9 (100.0)	91.2 (79.3)	99.5 (98.9)
R/R_free _(%)^b^		18.5/21.9	16/19.2
R.m.s.d. bonds (Å)		0.013	0.009
R.m.s.d. angles (°)		1.391	1.420
Ramachandran plot (%)			
Most favoured		92.8	92.8
Allowed		7.2	7.2

### Dimer formation

The considerable contact area between some symmetry related molecules in the crystal packing of both crystal forms suggested that AFV3-109 might be a dimer. Strengthening this argument, identical dimers could be formed from symmetry related molecules in both crystal forms. An r.m.s. deviation of 0.51 Å is observed for all Cα atoms of the two dimers. The dimer association creates a dyad-symmetric ten stranded half β-barrel, forming a curved 10 stranded cradle that enrols the two loops connecting strands β1 and β2 (Figure 1b,c). Dimer formation mainly packs the side of the β-sheet that is covered by loops and involves an extended surface area (1432 Å^2^), corresponding to 22% of the accessible surface area of the monomer. This important fraction of the accessible surface that is buried strongly suggests that this association corresponds to a biologically significant dimer [24]. We confirmed dimer formation in solution both by gel filtration chromatography and by gluteraldehyde cross linking experiments coupled to SDS PAGE analysis (results not shown).

The dimer is stabilised by three types of interactions. First, the main dimer contact is provided by extended anti-parallel β-sheet formation between the β2 strands from both subunits (illustrated in Figure 1b,c). Secondly, the loop connecting β1 and β2 forms a short anti parallel stretch (residues 8 to 10). Third, some hydrogen bonds are formed between well conserved side chains from the loops connecting β3/α2 and β4/α3. Overall, 24 H-bonds and numerous VDW contacts are observed between the monomers.

### Dimer cavity and glycerol binding site

The cradle encloses a big cavity at the centre of the dimer, whose floor is made up by the β1β2 loops that are also involved in dimer formation. This cavity, with an estimated volume of 1950 Å^3 ^is covered by parts of loops L3 (residues 43 to 47, between β3 and α2) and the region comprising residues 81 to 93. Many conserved residues line the cavity (see further), which is filled by well identified waters (about 50 were identified in the 1.3Å structure).

**Figure 2:**
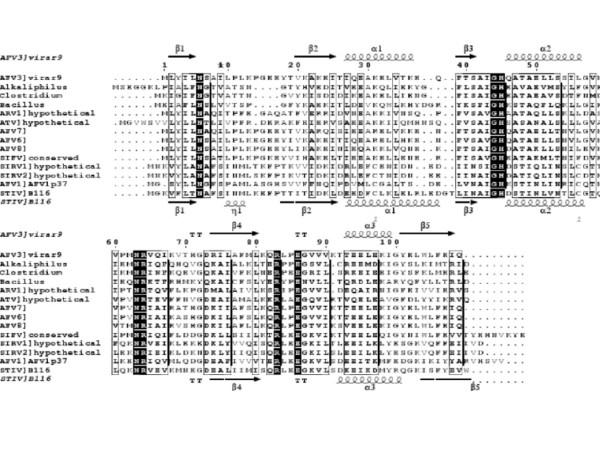
Sequence alignment of AFV3-109 orthologs, containing archaeviral and gram positive bacterial sequences. Secondary structure elements as extracted from the AFV3-109 and B116 structures are shown above and beneath the aligned sequences. Figure generated with ESPript [38].

**Figure 3:**
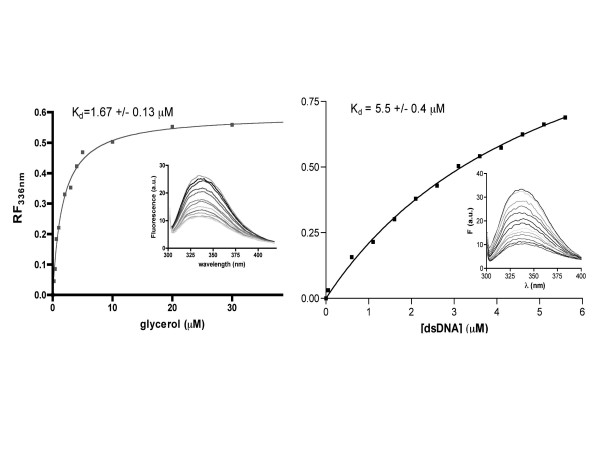
Tryptophan fluorescence quenching study on AFV3-109. The tryptophan fluorescence quenching titration of AFV3-109 at 336 nm emission wavelength (expressed as 1-I/I_0_, with I_0 _the intensity of fluorescence in absence of quencher and I the intensity of fluorescence upon addition of quencher). Titration with glycerol (A) and dsDNA (B). The K_Dapp _values are calculated from the fitting of the data to a single exponential (GraphPad). *Inset*, fluorescence emission spectra in the presence of increasing concentrations of glycerol or dsDNA.

The refinement of the C222_1 _crystal structure identified the presence of a strong residual electron density in the cavity, in close contact with the His45 side-chain (Figure 1f). We modelled this density as a glycerol molecule, present in the mother liquid as crystal cryoprotectant. The glycerol model could be well refined in the C222_1 _space group, with a final B-factor of 14.6 Å^2^. This B factor is similar to those of the surrounding residues, showing that glycerol is well defined in the structure. In contrast, a glycerol molecule could not fit into a residual density present near His45 in the P3_1_21 space group. The glycerol moiety forms strong hydrogen bonds with Asn6 Oδ 1, Val90 N and Glu86 Oε1 from the second monomer. It is also engaged in van der Waals interactions with the Leu80 and Thr48 side chains, and stacks against His45 imidazole ring. All the residues of the glycerol binding site are totally conserved in the sequences of viral AFV3-109 homologues (Figure 2). The conserved nature of this pocket suggests that it may be a biding site of functional relevance. In order to further verify interactions with glycerol, we have characterised its binding in solution by fluorescence titration (Figure 3a). Glycerol causes quenching of the protein fluorescence. Titration of this signal yielded a binding constant of 1.7 μM.

### Comparison with STIV B116 protein

A search for structural homologues using the msd server at the EBI (Hinxton, UK) identified the B116 protein of the Sulfolobus turreted icosahedral virus (STIV B116, PDB accession number 2BLK). As illustrated in Figure 1d, the monomers of AFV3-109 and B116 superpose with a r.m.s. deviation of 1.93Å for 93 Cα positions. AFV3-109 and B116 share 37% sequence identity and 55% sequence similarity. Interestingly, B116 crystallized with two copies in the asymmetric unit related by a non-crystallographic two fold axis. The resulting dimer arrangement is identical to that observed for the crystallographic AFV3-109 dimer, strengthening the argument that this arrangement corresponds to a genuine dimer in solution. Despite their identical fold, AFV3-109 and B116 present a marked difference within the long connection between β4 and β5 (comprising residues Leu80 to Gly100). While part of this loop closes the cavity in AFV3-109, it has completely swung out from the centre in the B116 dimer (Figure 1d). The different orientations of this connection in AFV3-109 and B116 are essentially caused by different main-chain dihedral angles in the ultimate residue preceding β5 (Gly100 and Lys109, respectively) that provokes a tilt of the α3 helix in B116 compared to AFV3-109. The residues at the tip of this connecting domain in superposed AFV3-109 and B116 are at a distance of 19.4 Å, while they contribute to dimer contacts in AFV3-109. For instance Gly87 Cα positions of the two AFV3-109 monomers are at 4.3 Å, while for B116 the homologous Gly93 Cα positions from the two monomers are at 37.8 Å. The β4-β5 connection forms the vault of the dome in the large central cavity of the AFV3-109 dimer. Due to its different configuration, this connection in B116 no longer seals the central cavity but rather walls a large groove.

B116 has a few insertions compared to AFV3-109. The first one, 4 residues long, is in the connection between β2 and β3 and results in one more turn in helix α2. The second (2 residues) is in the tip of the long connection between β4 and β5 and is juxtaposed to the best conserved sequence region (see further). This large conformational difference in the connection between β4 and β5 with B116 is obeyed for the two crystal forms of AFV3-109 who superpose perfectly in this region. In absence of clear functional data we cannot be certain whether this connection has intrinsic mobility or whether this is an inherent structural difference between the two orthologs. The B-factors within this connection in AFV3-109 are comparable to those of the remaining part of the structure. In B116 the β4-β5 connection of one of the two monomers present in the asymmetric unit is involved in crystal contacts with a neighbouring molecule. However, both monomers adopt the "open" conformation which therefore does not seem to be dictated by crystal packing forces. We suspect that B116 and AFV3-109 may exist in both the open and closed forms, and that the B116 dimer was trapped in the open conformation.

### AFV3-109 orthologs

AFV3-109 belongs to the most prominent clusters of apparent orthologs in crenarchaeal viruses. Besides STIV, homologs are clearly identified on genomes of AFV1, SIRV1/2 and ATV, representing 3 different viral families (Rudiviridae, Lipotrixviridae, Bicaudaviridae). Orthologs of unknown function are also present in a few gram positive bacteria: *Bacillus subtilis *(*yddF*), *Alkaliphilus metalliredigenes *and *Clostridium beijerincki*. AFV3-109 may be the strongest case of horizontal gene transfer among unrelated crenarchaeal virus families and between crenarchaeal viruses and bacteria. Its strong conservation in the crenarchaeal virus families allows us to analyze amino acid sequence conservation against structural data. Figure 2 shows the sequence alignment of the AFV3-109 orthologs with the superposed secondary structure elements as extracted from the AFV3-109 and B116 crystal structures. The best conserved sequence stretch, centred on His45, is contained within the long connection between β3 and β4. This residue is in close contact with Arg64 which also forms a hydrogen bond with carbonyl oxygen of the conserved Gly44. His45 Nδ 1 is hydrogen bonding with the totally conserved Glu86 carboxylate of the opposing monomer. The totally conserved Arg83 makes a dimer contact by hydrogen bonding with the Ala42 carbonyl group. These latter dimer interactions are absent in the much open form of B116.

### AFV3-109 as a transcription factor?

The AFV3-109 dimer has an interesting resemblance with the DNA binding modules (DBM) of transcription factors present in eukaryotic viruses (Figure 1e)[25,26]. The DBMs of the papilloma virus E2 protein and the Epstein-Barr Virus Origin binding protein EBNA1 can be superposed onto AFV3-109 with a r.m.s. of 3.05 and 2.85 Å respectively (on 40 and 44 Cα positions used in the alignment). The topology of AFV3-109 being different from that of the E2 and EBNA1 DBMs, however, a common evolutionary origin should be excluded. E2 and EBNA1 both bind palindromic DNA sequences by virtue of symmetrically disposed helices, although both proteins use very different strategies [25,26]. DNA binding modules are usually characterized by strong positively charged surface patches that interact with the negatively charged DNA phosphate backbone. As illustrated in Figure 1g, a strong positively charged surface patch is clearly absent in AFV3-109, which is on the contrary marked by a very negatively charged surface patch situated at the opposite of the dome of the cavity. However, a positive patch is observed inside the cavity, not accessible to outside ligands in AFV3-109 due to the closed configuration of the β4β5 connection. Modelling AFV3-109 in an open conformation based on that of its B116 ortholog (Figure 1h) reveals that the positively charged surface patch becomes available for external interactions. In order to test the hypothesis about an eventual role in transcription, we tested whether AFV3-109 could bind DNA *in vitro*. As shown by fluorescence quenching titrations, dsDNA binds to AFV3-109 with a Kapp of 5 μM (Figure 3b). The biological significance of this interaction remains to be determined.

### *AFV3-109 *is part of a proviral gene cluster present in some Firmicutes

**Figure 4:**
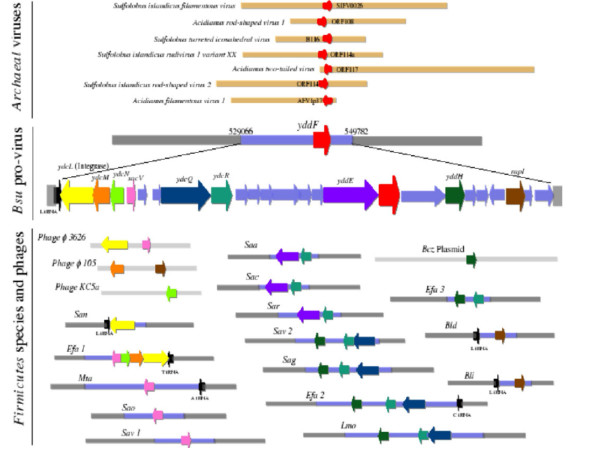
Genomic context of the AFV3-109 ortholog in Firmicutes. The *yddF *gene (big red arrow) from *Bacillus subtilis *has homologues in several genomes of Archaeal viruses (small red arrows), such as: the SFV0026 gene in *Sulfolobus islandicus filamentous virus*, the ORF108 in *Acidianus rod-shaped virus 1*, the B116 gene in *Sulfolobus turreted icosahedral virus*, the ORF114a in *Sulfolobus islandicus rudivirus 1 variant XX*, the ORF117 in *Acidianus two-tailed virus*, the ORF114 in *Sulfolobus islandicus rod-shaped virus 2*, and the AFV1p37 gene in *Acidianus filamentous virus 1*. The *yddF *gene is found inside an integrated pro-virus in the *Bacillus subtilis *genome. This pro-virus starts with the *ydcL *gene (yellow arrow), which codes for an integrase and is next to a leucine tRNA gene (black arrow). The *ydcL *and *sacV *(pink arrow) genes have homologues in the *Clostridium phage *φ *36262 *genome; the *ydcM *(orange arrow) and *rapI *(brown arrow) genes have homologues in the *Bacillus phage *φ *105 *genome, the *ydcN *(light green arrow) gene in the *Lactobacillus gasseri phage KC5a*, and the *ydcN *(light green arrow) gene in the *Bacillus cerus E33L *Plasmid *pE33L466*. Nine out of the twenty-four genes in this pro-virus: *ydcL*, *ydcM*, *ydcN*, *sacV*, *ydcQ *(dark blue arrow), *ydcR *(blue green arrow), *yddE *(purple arrow), *yddH *(dark green arrow), and *rapI*, have homologues in other probable integrated pro-viruses (light blue rectangles; the grey rectangles represent the genome genes outside the probable inserted pro-virus) in several genomes of Firmicutes. The names of the species have the following annotation: *B.su *is * Bacillus subtilis*, *B.cz *is * Bacillus cerus *E33L, *B.ld *is * Bacillus licheniformis *ATCC 14580, *B.li *is *Bacillus licheniformis *DSM13, *E.fa *is *Enterococcus faecalis*, *L.mo *is *Listeria monocytogenes *EGD-e, *M.ta *is *Moorella thermoacetica*, *S.ao *is *Staphylococcus aureus *NCTC8325, *S.av *is *Staphylococcus aureus *Mu50, *S.aa *is *Staphylococcus aureus *USA300, *S.ac *is *Staphylococcus aureus *COL, *S.ar *is *Staphylococcus aureus *MRSA252, *S.an *is *Streptococcus agalactiae *NEM316 (serotype III), and *S.ag *is *S.agalactiae *2603 (serotype V). The size and the genomic location of the probable integrated pro-virus is also indicated.

Most proteins encoded by viruses from hyperthermophilic crenarchaea are orphan or only present in viruses of the same family. AFV3-109 is the most conserved protein in different families of crenarchaeal viruses. Interestingly, we noticed that, unlike all other proteins from crenarchaeal viruses, AFV3-109 has three closely related homologues in several bacterial genomes: the *AmetDRAFT_3039 *gene in *Alkaliphilus metalliredigenes*, the *CbeiDRAFT_4464 *gene in *Clostridium beijerincki*, and the *yddF *gene in *Bacillus subtilis*. We wonder if these bacterial homologs could be also of viral origin. Unfortunately, only the *B. subtilis *genome was available for further detailed analysis. Interestingly, we found that the *yddF *gene is present in a twenty-four-gene cluster (Figure 4) whose nucleotide composition is significantly different from the rest of the genome. This gene cluster begins with a gene coding for an integrase, which is found next to a leucine tRNA gene, suggesting that it corresponds indeed to an integrated provirus. Blast search revealed that fourteen genes did not exhibit any significant matches with other proteins, six genes have homologues in phage genomes or plasmids, and nine genes have homologues in several genomes of Firmicutes. We then decided to analyze the genomic regions containing these homologous genes in the complete sequenced genomes of Firmicutes. All the analyzed genes are also found in gene clusters displaying significant atypical nucleotide compositions, some of which being adjacent tRNA genes (Figure 4). These gene clusters also contain genes having homologs in several phage and Firmicutes genomes, however the majority of analyzed genes are orphans. All these evidences suggest that the *yddF *gene is inside a twenty-four-gene long pro-virus integrated in the *B. subtilis *genome (genomic location: 529066 – 549782), that belongs to a widespread family of viruses infecting Firmicutes species. Although it has been known for some time that head and tailed viruses from archaea encode a few genes with bacterial homologs, the AFV3-109/YddF protein family is the first example of a protein family with members present in both bacterial and crenarchaeal viruses. This observation, together with the already noticed unusual conservation of AFV3-109 among different families of crenarchaeal viruses highlights the importance of this protein. It could be explained either by an unusual gene transfer between archaeal and bacterial viruses or it could be another example of an ancient protein that was present in an ancient viral world predating the separation of archaea and bacteria [27,28].

### Concluding remarks

It was previously recognised that the structural analysis of virus coat proteins might prove extremely valuable to establish evolutionary relationships between viruses that infect various hosts. In this paper we present the crystal structure of the best conserved ORF among crenarchaeal viruses, showing that it has a unique architecture, shared by its STIV ortholog B116. The identification of a highly conserved surface patch on the molecular surface will offer the opportunity to test functional hypothesis through site directed mutagenesis.

## Materials and methods

### Cloning, expression, purification

AFV3-109 was amplified by PCR using genomic DNA as a template. An additional sequence coding for a 6 histidine tag was introduced at the 3' end of the gene during amplification. The PCR product was then cloned into a derivative of pET9 vector. Expression was done at 37°C overnight using the transformed *E. coli *Gold(DE3) strain and 2xYT medium (BIO101 Inc.). When the cell culture reached an OD600nm of 1, protein expression was induced with 0.5 mM IPTG (Sigma) and the cells were grown for a further 4 hours. Cells were harvested by centrifugation and resuspended either in 40 ml of 20 mM Tris-HCl pH 7.5, 200 mM NaCl, 5 mM β-mercaptoethanol (C222_1 _space group) or in 20 mM Na Citrate pH 5.6, 200 mM NaCl, 5 mM β-mercaptoethanol (P3_1_21 space group). Then cells were stored overnight at -20°C. Cell lysis was completed by sonication. The His-tagged protein was purified on a Ni-NTA column (Qiagen Inc.), eluted with imidazole and loaded on to a Superdex75 column (Amersham Pharmacia Biotech), equilibrated against either 20 mM Tris-HCl pH7.5, 200 mM NaCl, 10 mM β-mercaptoethanol (C222_1 _space group) or 20 mM Na Citrate pH 5.6, 200 mM NaCl, 10 mM β-mercaptoethanol (P3_1_21 space group). Selenomethionine-substituted AFV3-109 was produced and purified as the native protein (P3_1_21 space group). The homogeneity of the proteins was checked by SDS-PAGE, and the SeMet labelling by mass spectrometry.

### Structure resolution

AFV3-109 native crystals were grown from a 1:1μl mixture of protein (8 mg/ml, purified at pH 5.6) with 30% PEG4000, 0.2 M NaAc, pH 4, and from a 1:1μl of protein (16 mg/ml, purified at pH 7.5) with 25% PEG 4000, 0.1 M NaAc, 0.1 M tris-HCl pH 8.8, 15% glycerol. SeMet substituted AFV3-109 crystals were grown from a 0.5:0.5μl mixture of protein (14 mg/ml, purified at pH 5.6) with 30% PEG 4000, 0.2 M NaAc, pH 4. Crystals were grown using the hanging drop method at 20°C. For cryoprotection, crystals were soaked in a mixture of precipitant solution with 30% glycerol. Crystals were then flash frozen at 100 K.

X-ray diffraction data from a crystal of the SeMet substituted AFV3-109 were collected on beamline BM30A (ESRF) at the Se *K*-edge. The crystals diffracted to 2Å and belong to space group P3_1_21 with one molecule per asymmetric unit, corresponding to 49.5% solvent content. Native data of AFV3-109 from crystals grown at pH 4.0 were collected on the ID14-1 beamline (ESRF) to 1.3Å resolution. Crystals grown at pH 8.8 diffracted to 1.5Å and belong to space group C222_1 _with one molecule per asymmetric unit, corresponding to 45% solvent content. Data processing was carried out with the program MOSFLM [29] and scaling and merging with SCALA [30].

The structure of AFV3-109 was determined using SAD X-ray diffraction data, collected from a Se-Met labeled crystal at 2Å resolution. Three Selenium atom sites were found with the program SHELXD in the 50-2 Å resolution range [31]. These sites were used for phasing with the program SOLVE [32]. After solvent flattening with the program RESOLVE, the quality of the electron density map allowed automated construction of ~90% of the model. This partial model was then refined against the 1.3Å data set with the Arp/Warp program [33] that allowed automated building of the missing residues. The structure was refined with REFMAC [34] and the model manually corrected using the Turbo molecular graphics program [35]. All the residues (from Met1 to Gln109 and 284 water molecules) are well defined in electron density map and fall within the allowed regions of the Ramachandran plot, as defined by the program PROCHECK [36].

The structure of AFV3-109 in space group C222_1 _was solved by molecular replacement with the structure in P3_1_21 space group using the program MOLREP [37]. At the end of the refinement there was some clear residual electron density for a bound glycerol. Some residual density was present at the same site of the protein in the P3_1_21 space group, but did not allow fitting a glycerol moiety into. Statistics for all the data collections and refinement of the different structures are summarized in Table 1.

### Fluorescence quenching experiments

Fluorescence quenching of the single tryptophan in AFV3-109 was measured by using a Cary Eclipse (Varian) equipped with a front-face fluorescence accessory at 20°C, by using 5-nm excitation and 10-nm emission bandwidths. The excitation wavelength was 290 nm and the emission spectra were measured between 300 and 410 nm. Titrations were performed in a 1-ml quartz fluorescence cuvette containing 1 μM protein in 10 mM Tris-HCl buffer, 200 mM NaCl, 10 mM β-mercaptoethanol, pH 7.5, and by the successive addition of aliquots of glycerol or dsDNA stock solution. Data were analyzed by plotting the relative fluorescence intensities at 336 nm at increasing concentrations of quencher. Dissociation equilibrium constant (*K*_Dapp_) values were determined from data fitted to a single exponential equation, by using the PRISM 4 nonlinear regression tool (GraphPad, San Diego).
